# Supplementary material for: Adapting the marine stewardship council’s risk-based framework to assess the impact of towed bottom fishing gear on blue carbon habitats
Source: PLoS One. 2023 Nov 16;18(11):e0288484. doi: 10.1371/journal.pone.0288484 (PMC10653409; doi:10.1371/journal.pone.0288484)
Supplement: S1 File — (DOCX) [file pone.0288484.s001.docx]

# Annex’s

# Annex 1: MSC RBF CSA scoring attributes

Table 4: Consequence and Spatial Analysis (CSA) attributes description from the Marine Stewardship Council (MSC) Risk Based Framework (RBF) (taken from MSC FCP v2.2. and associated RBF excel scoring workbook)

| **Consequence Spatial Analysis (CSA) Score (scores 1.18-4.24)** | | |
| --- | --- | --- |
| Consequence Score (scores 1-3) | | Spatial Score (scores 0.63-3) |
| Habitat-productivity attributes (scores 1-3) | Gear-habitat interaction attributes (scores 1-3) | Spatial attributes (scores 0.5-3) |
| Regeneration of biota:  *Scored on the basis of the rate of the recovery of biota associated with the habitat using information on age, growth, and recolonisation of biota where available (MSC FCP v2.2 Table PF12).*  *Scores 1-3* | Removability of biota:  *Scored on the basis of the likelihood of attached biota being removed or killed by interactions with fishing gear (MSC FCP v2.2 Table PF14).*  *Scores 1-3* | Gear footprint:  *Scored on the basis of the gear’s potential for disturbance and the number of encounters required to produce an impact on a habitat, taking into account the size, weight, and mobility of individual gears and the footprint of the gears (MSC FCP v2.2 Table PF16).*  *Scores 1-3* |
| Natural disturbance:  *Scored on the basis of the natural disturbance that is assumed to occur at the particular depth zone in which the habitat and fishing activity occurs (MSC FCP v2.2. Table PF13).*  *Scores 1-3* | Removability of substratum:  *Scored on the basis of clast size and likelihood of the substratum being moved (MSC FCP v2.2 Table PF14).*  *Scores 1-3* | Spatial overlap:  *Scored on the basis of spatial overlap between* *the habitat(s) distribution within the relevant “managed area” and the distribution of areas fished by the fishery (MSC FCP v2.2 Table PF17).*  *Scores 0.5-3* |
|  | Substratum hardness:  *Scored on the basis of substrata composition (MSC FCP v2.2 Table PF15).*  *Scores 1-3* | Encounter-ability:  *Scored on the basis of the likelihood that a fishing gear will encounter the habitat within the “managed area”, taking into account the nature and deployment of the fishing gear and the possibility of its interaction with the habitat (MSC FCP v2.2 Table PF17).*  *Scores 0.5-3* |
|  | Substratum ruggedness:  *Scored on the basis of the extent to which available habitat is actually accessible to mobile gear given the ruggedness of the substratum (MSC FCP v2.2 Table PF15).*  *Scores 1-3* |  |
|  | Seabed slope:  *Scored on the basis of the impact to habitat that occurs as a result of slope steepness and mobility of substrata once dislodged (MSC FCP v2.2 Table PF15). Scoring this attribute shall consider the degree of slope.*  *Scores 1-3* |  |

# Annex 2: Scoring Elements (Southern North Sea case study)

Table 5: Habitat types present in the southern North Sea case study. The European Nature Information System (EUNIS) Habitat classifications is used to define each habitat type for inclusion in the Consequence Spatial Analysis (CSA) carbon extension.

*For the purpose of the CSA Carbon extension littoral rock is viewed as macroalgae substrate and distribution with 100% coverage

| Scoring element | Habitat classification | Description | EUNIS Level 3 | Substratum | Biota | Biome (depth meters (m)) |
| --- | --- | --- | --- | --- | --- | --- |
| 1 | Littoral rock and other hard substrata* | Rocky habitats - suitable substrate for kelp and macroalgae:  Moderate energy littoral rock; Low energy littoral rock; Features of littoral rock | A1  A1.1  A1.1+A1.2  A1.1+A1.4  A1.2  A1.2+A1.4  A1.3  A1.4  A3  A3.1  A3.1+A4.1  A3.1+A4.2  A3.1+A5.1  A3.1+A5.4  A3.2  A3.2+A4.1+A4.2  A3.2+A4.2  A3.2+A5.4  A3.3  A3.7  A3.7+A4.1+A5.2  A4  A4.1  A4.2  A4.3 | Igneous, metamorphic, or sedimentary bedrock (>3 m) | Small erect/ encrusting Dominated by: *Laminaria digitata* | Coast (0-25m) |
| 2 | Littoral coarse sediment | Littoral coarse sediments include shores of mobile pebbles, cobbles and gravel, sometimes with varying amounts of coarse sand. | A2.1  A2.1+A2.2  A2.1+A2.4 | Fine - Coarse Sediment (1-4mm) | Small burrowing dominated by: Infaunal bioturbators | Coast (0-25m) |
| 3 | Littoral sand, muddy sand | Shores comprising clean sands (coarse, medium or fine-grained) and muddy sands with up to 25% silt and clay fraction. (A2.2) | A2.2  A2.2 + A2.3 | Fine Mud (0.1mm) & sand (0.1-1mm) | Small burrowing dominated by: Infaunal bioturbators | Coast (0-25m) |
| 4 | Littoral mud | Shores of fine particulate sediment, mostly in the silt and clay fraction (particle size less than 0.063 mm in diameter), though sandy mud may contain up to 40% sand (mostly very fine and fine sand). | A2.3  A2.3+A2.4  A2.3+A2.5  A2.3+A2.8 | Fine mud (0.1mm) | Small burrowing dominated by: Infaunal bioturbators | Coast (0-25m) |
| 5 | Littoral mixed sediments | Shores of mixed sediments ranging from muds with gravel and sand components to mixed sediments with pebbles, gravels, sands and mud in more even proportions. (A2.4) | A2  A2.4  A2.4+A2.8 | Fine Mud (0.1mm) & sand (0.1-1mm) | Small burrowing dominated by: Infaunal bioturbators | Coast (0-25m) |
| 6 | Coastal saltmarshes and saline reedbeds | Angiosperm-dominated stands of vegetation, occurring on the extreme upper shore of sheltered coasts and periodically covered by high tides. | A2.5 | Biogenic origin | Flora dominated by: Saltmarsh and reedbeds. | Coast (0-25m) |
| 7 | Littoral sediments dominated by aquatic angiosperms | Dominants are *Zostera* spp. A2.6 & A5.5 seaweeds A2.8 mixed sediment (intertidal seagrass beds) | A2.6;  A2.8;  A5.5 | Biogenic origin | Flora dominated by: Seagrass species (*Zostera spp*). | Coast (0-25m) |
| 8 | Sublittoral coarse sediment | Coarse sediments including coarse sand, gravel, pebbles, shingle and cobbles which are often unstable due to tidal currents and/or wave action | A5.1 | Fine (coarse sediments 1-4mm) | Small burrowing dominated by: Infaunal bioturbators | Shelf (25-200m) |
| 9 | Sublittoral sand | Clean medium to fine sands or non-cohesive slightly muddy sands on open coasts, offshore or in estuaries and marine inlets. | A5.2 | Fine Mud (0.1mm) & sand (0.1-1mm) | Small burrowing dominated by: Infaunal bioturbators | Shelf (25-200m) |
| 10 | Sublittoral mud | Sublittoral mud and cohesive sandy mud extending from the extreme lower shore to offshore, circalittoral habitats. | A5.3 | Fine Mud (0.1mm) & sand (0.1-1mm) | Small burrowing dominated by: Infaunal bioturbators | Shelf (25-200m) |
| 11 | Sublittoral mixed sediments | Sublittoral mixed (heterogeneous) sediments found from the extreme low water mark to deep offshore circalittoral habitats | A5.4  A5.4 + A5.1  A5 | Fine Mud (0.1mm) & sand (0.1-1mm) | Small burrowing dominated by: Infaunal bioturbators | Shelf (25-200m) |
| 12 | Biogenic reefs | Biogenic *Sabellaria spp.* reef | A2.7  A5.6 | Biogenic origin | Small burrowing dominated by burrowing reef worms, *Sabellaria spp.* | Shelf (25-200m) |

# Annex 3: Mobile Bottom-contacting Gear

Table 6: Glossary terms and BENTHIS métier groupings used to define higher level métier groupings. As presented in (38).

| Surface | < 2 cm penetration depth of the gear components. * *Not used in this assessment* |
| --- | --- |
| Subsurface | ≥2 cm penetration depth of the gear components. |
| Beam trawl (TBB) | For beam trawls (TBBs) the footprint consists of two components: (i) the shoes of the beam, and (ii) the ground gear. Before that part of the footprint is made by the tickler chains of the trawl, if such chains are deployed. |
| Dredge (DRB) | For dredges (DRBs) the ground gear component defines the footprint which is homogeneous across the entire width of the dredge, even if teeth are used. |
| Demersal Seine (DS) | For seines (DSs) two main types of footprint occur: (i) from the seine rope, and (ii) from the seine ground gear. |
| Otter Trawl (OT) | For otter trawls (OTs), the footprint is composed of (i) the otter boards, (ii) the sweeps, and (iii) the trawl ground gear. |
| BENTHIS Métier | 14 standard BENTHIS métier groupings (see below) that have similar gear footprints, and which can be aggregated up to describe higher level gear groupings (beam, dredge, demersal seine, otter trawl) |
| OT_CRU | Otter trawl for Nephrops or shrimp |
| OT_DMF | Otter trawl for cod or plaice |
| OT_MIX | Otter trawl for other species |
| OT_MIX_CRU | Otter trawl for mixture of species with focus on shrimp (note: no data were submitted for this gear category) |
| OT_MIX_DMF_BEN | Otter trawl for mixed benthic fish |
| OT_MIX_DMF_PEL | Otter trawl for bentho-pelagic fish (note: no data were submitted for this gear category) |
| OT_MIX_CRU_DMF | Otter trawl for Nephrops and mixed fish |
| OT_SPF | Otter trawl for sprat or sandeel |
| TBB_CRU | Bottom trawl for crangon |
| TBB_DMF | Bottom trawl for sole and plaice |
| TBB_MOL | Bottom trawl for molluscs |
| DRB_MOL | Dredge for scallops and mussels |
| SDN_DMF | Danish seine for plaice and cod (note: there is no subsurface component for this gear) |
| SSC_DMF | Scottish seine for cod, haddock, and other flatfish |

# Annex 4: SAR attribute implementation

| Spatial attribute | Score | | | | | |
| --- | --- | --- | --- | --- | --- | --- |
|  | 0.5 | 1 | 1.5 | 2 | 2.5 | 3 |
| Encounter-ability | ≤15% | ≤30% | ≤45% | ≤60% | ≤75% | >75% |
| Swept Area Ratio (SAR) | ≤0.004 | ≤0.03 | ≤0.10 | ≤0.23 | ≤0.57 | >0.57 |

Table 7: Swept Area Ratio converted into scores forMSC Ecounterability scores . Adapted from MSC FCP V2.2 Table PF17.

Figure 5: Swept Area Ratio (SAR) ranges for the United Kingdoms (UK) Economic Exclusion Zone (EEZ) (CSA scores awarded ≤0.004 = 0.5, ≤0.03 = 1, ≤0.10 = 1.5, ≤0.23 = 2, ≤0.57 = 2.5, >0.57 = 3).

Figure 6: Sub-surface Swept Area Ratio (SAR) distribution analysis to determine the SAR ranges used in CSA Carbon extension scoring. Total number of c-squares in the United Economic Exclusion Zone with SAR values (32115) was divided into six to provide SAR scoring ranges. SAR values are indicated at the top of each colloumn (CSA scores awarded ≤0.004 = 0.5, ≤0.03 = 1, ≤0.10 = 1.5, ≤0.23 = 2, ≤0.57 = 2.5, >0.57 = 3).

# Annex 5: Carbon attribute estimates

OC stock and accumulation estimates vary depending on the literature sources used. The data synthesised in this study provide observations of OC at <10cm depth but do not support an estimate of OC stock and accumulation at >10cm without extrapolation. Extrapolation introduces uncertainty in the OC stock and accumulation estimates at >10cm. Additionally, the maximum penetration depth recorded for mobile gears (used in the UK) is ≤35 cm (in mud) (67). This means a 10cm OC stock or accumulation estimate is likely to underestimate the vulnerability of OC and conversely a 1m profile an overestimate. A CSA carbon extension was run with OC stock and accumulation estimates assuming a gear penetration of both 0.1 and 1m sediment depths to understand differences in risk assessment outcomes. Because OC stock and accumulation values are extrapolated using equal distribution across the depth profile, OC stock and accumulation attribute scores were similar and therefore the overall CSA score unchanged (see supplementary material).

The conservative 1m OC stock and accumulation estimates were used in the final CSA carbon extension.

Table 8: Organic carbon (OC) stock estimates (kgC/m^-2^). Stock estimates apply to the United Kingdom (UK) economic exclusion zone (EEZ) only. Estimates in **bold** were used to assign the CSA carbon extension carbon stock attribute scores for each habitat type (scoring element). Where multiple and conflicting values are presented, this study takes a new average of the averages. The original value estimates are presented as seen in source documents, along with any primary and secondary literature review references. N/A = Not Applicable. *Signifies noticeable outliers and excluded values from this study’s average calculations.

| Habitat classification | EUNIS Level 3 Code | Habitat component | OC stock kgC/m^-2^ 1m depth | | | Study area | Original references | Literature review reference |
| --- | --- | --- | --- | --- | --- | --- | --- | --- |
|  |  |  | Min | Max | Average |  |  |  |
| Rocky habitats | A1 | Vegetative OC Intertidal | 0.24 | 1.65 | 0.95 | Central and North Wales | (68) | (14) |
|  |  | Vegetative OC Subtidal | 0.22 | 1.22 | 0.72 | UK | (69) | (14) |
|  |  |  | 0.08 | 0.28 | 0.18 | UK | (70) | (14) |
|  |  | Vegetative OC Subtidal -warm | 0.01 | 0.14 | 0.08 | UK | (49) | (44) |
|  |  | Vegetative OC Subtidal -cold | 0.07 | 1.2 | 0.64 | UK | (49) | (44) |
|  |  | **Vegetative study averages** | **N/A** | **N/A** | **0.51** | **UK** | **This study** | **This study** |
| Littoral sand, muddy sand | A2.2 | **Sediment OC** | **1.30** | **18.60** | **6.50** | **UK** | (71) | (44) |
|  |  | Sediment OC | 1.30 | 18.60 | 6.50 | UK | (71) | (14) |
| Littoral mud | A2.3 | **Sediment OC** | **5.40** | **35.60** | **19.90** | **UK** | (72–75) | (14,44) |
|  |  | Vegetative OC | 0.05 | 0.52 | 0.29 | Southwest England | (76) | (14) |
|  |  | Vegetative OC | 0.00 | 0.13 | 0.07 | UK wide | (77–79) | (14,80) |
|  |  | Sediment OC (state Natural) | 13.20 | 31.60 | 22.40 | UK | (45,72,77,81–84) | (44) |
|  |  | Sediment OC (state Restored) | 10.10 | 25.00 | 17.55 | UK | (45,72,77,81–84) | (44) |
|  |  | Sediment OC (state not designated) | 12.70 | 69.00 | 40.85 | UK | (45,72,77,81–84) | (44) |
|  |  | **Vegetative + Sediment study averages** | **N/A** | **N/A** | **27.11** | **UK** | **This study** | **This study** |
| Littoral sediments dominated by aquatic angiosperms | A2.6 | Vegetative OC | 0.01 | 0.05 | 0.03 | Southwest england | (85) | This study |
|  |  | Sediment OC | 4.6 | 38.00 | 21.30 | UK | (73,86,87) | (44) |
|  |  | **Vegetative + Sediment study averages** | **N/A** | **N/A** | **21.33** | **UK** | **This study** | **This study** |
| Sublittoral sand | A5.2 | Sediment OC | 0.40 | 7.60 | 1.70 | UK | Multiple Cefas surveys | (44) |
|  |  | Sediment OC | 0.50 | 2.60 | 1.60 | UK | (45) | (14) |
|  |  | Sediment OC | 0.40 | 7.60 | 1.80 | UK | (20,47) | (14) |
|  |  | **Sediment OC** | **N/A** | **N/A** | **1.70** | **UK** | **This study** | **This study** |
| Sublittoral mud | A5.3 | Sediment OC | 0.60 | 12.30 | 5.50 | UK | Multiple Cefas surveys | (44) |
|  |  | Sediment OC | 0.60 | 12.30 | 5.50 | UK | (20,47) | (14) |
|  |  | Sediment OC | 39.0* | 208.0* | 123.5* | UK | (45) | (14) |
|  |  | **Sediment OC** | **N/A** | **N/A** | **5.50** | **UK** | **This study** | **This study** |

Table 9: Organic carbon (OC) accumulation estimates (kgC/m^-2^/yr^-1^). Accumulation estimates apply to the United Kingdom (UK) economic exclusion zone (EEZ) only. Estimates in **bold** were used to assign the CSA carbon extension carbon Accumulation attribute scores for each habitat type (scoring element). Where multiple and conflicting values are presented, this study takes a new average of the averages. References to the original value estimates are presented, along with any secondary literature review references. n.d = No Data available. N/A = Not Applicable

| Habitat classification | EUNIS Level 3 Code | Habitat component | OC Accumulation kgC/m^-2^/yr^-1^ | | | Study area | Original references | Literature review reference |
| --- | --- | --- | --- | --- | --- | --- | --- | --- |
|  |  |  | Min | Max | Average |  |  |  |
| Rocky habitats | A1 | Vegetative production intertidal | 0.125 | 0.727 | 0.378 | UK wide | (68–70) | (14) |
|  |  | Vegetative production subtidal | 0.151 | 0.570 | 0.301 | UK wide | (68–70) |  |
|  |  | **This study (intertidal and subtidal average)** | **N/A** | **N/A** | 0.340 | **UK** | **This study** | **This study** |
| Littoral sand, muddy sand | A2.2 | **Sediment** | **n.d** | **n.d** | **0.45** | **UK** | (71) | (44) |
| Littoral mud | A2.3 | Sediment | 0.073 | 0.094 | 0.084 | UK | (72) | (14) |
|  |  | **Sediment** | **0.073** | **0.094** | **0.084** | **UK** | (72) | (44) |
| Saltmarsh | A2.5 | Sediment OC (not specified) | 0.140 | 0.196 | 0.161 | UK wide | (72,76) | (44) |
|  |  | Sediment OC (state natural) | n.d | n.d | 0.119 | UK wide | (72,76) | (44) |
|  |  | Sediment OC (state restored) | 0.066 | 0.127 | 0.096 | UK wide | (72,76) | (44) |
|  |  | Vegetation production | 0.042 | 0.235 | 0.138 | Unknown | (88) assuming d/w 25% C | (14) |
|  |  | **This Study (vegetation + sediment)** | **N/A** | **N/A** | **0.263** | **UK** | **This study** | **This study** |
| Littoral sediments dominated by aquatic angiosperms | A2.6 | Sediment | 0.067 | 0.105 | 0.086 | non-UK sites | (89) | (44) |
|  |  | Vegetative production | n.d | n.d | 0.274 | unkown | from increase in dry mass of Zostera marina (90,91) | (14) |
|  |  | **This Study (Sediment + vegetative production)** | **N/A** | **N/A** | **0.360** | **UK and Non-uk sites** | **This study** | **This Study** |
| Sublittoral sand | A5.2 | Sediment | 0.000 | 0.059 | 0.030 | UK wide | (92,93) | (44) |
|  |  | Sediment | 0.000 | 0.000 | 0.000 | UK wide | (45) | (14) |
|  |  | **This Study** | **N/A** | **N/A** | **0.030** | **UK wide** | **This study** | **This study** |
| Sublittoral mud | A5.3 | Sediment | nd | nd | 0.059 | South England | (93) | (14) |
|  |  | Sediment | 0.019 | 0.292 | 0.155 | Unknown | (45) | (14) |
|  |  | Sediment | 0.000 | 0.059 | 0.030 | Uk wide | (92,93) | (44) |
|  |  | **Sediment** | **N/A** | **N/A** | **0.044** | **UK wide** | **This study** | **This study** |

# Annex 6: CSA risk score

Table 10: MSC CSA scores for each habitat type (scoring element) in the southern North Sea case study. Habitats are presented in order of CSA risk score, lowest to highest.

| **Scoring element** | **Consequence score** | **Spatial score** | **CSA risk score** | **% of maximum CSA risk score (4.24)** | **Explanation of CSA risk score** |  |
| --- | --- | --- | --- | --- | --- | --- |
| Littoral rock and other hard substrata (A1.1) | 1.44 | 1.44 | 2.04 | 48.14 | Kelp and macroalgae have longer regeneration time compared to purely sedimentary habitats, but the hard substrate means all other habitat productivity attributes are scored lower. Combined with relatively low spatial attribute scores, the overall CSA score is the lowest of all assessed habitat types. |  |
| Littoral coarse sediment (A2.1) | 1.89 | 0.91 | 2.10 | 49.43 | Littoral coarse sediments have lower regeneration times, but the alterable substrate makes it more vulnerable to impacts, compared to hard rock, therefore it scores higher on the overall consequence score. It has a lower spatial score due to decreased spatial overall and SAR, but the combined CSA score is 1.29% higher than rock. |  |
| mixed sediments (A2.4) | 1.89 | 1.14 | 2.21 | 52.09 | Mixed sediments consequence attributes are scored the same as littoral coarse sediments, but the spatial overlap attribute score is 0.5 higher than littoral coarse sediment, raising the overall CSA score to 2.21, 2.66% higher than littoral coarse sediments. | |
| Littoral sand, muddy sand (A2.2) | 1.89 | 1.31 | 2.30 | 54.22 | Mixed sediments consequence attributes are scored the same as littoral coarse sediments and mixed sediments. The spatial overlap attribute is scored the same as mixed sediments, but the SAR attribute is higher. The overall CSA score is 2.13% higher than mixed sediment. |  |
| Littoral sediments dominated by aquatic angiosperms (A2.6) | 2.22 | 1.14 | 2.50 | 58.96 | Seagrasses have a regeneration time of decadal, resulting in the highest attribute score of 3 to be awarded. This raises the consequence score to one of the highest across all habitat types. Seagrasses however have one of the lowest spatial scores, resulting in a CSA risk of 2.40, which is 2.4% higher than littoral sand. | |
| Sublittoral mud (A5.3) | 2.11 | 1.65 | 2.68 | 63.21 | Sublittoral mud scores the same on the consequence attributes as inshore sedimentary habitats, except for the natural disturbance attribute. These habitats are further offshore and less disturbed naturally, resulting in the higher consequence score. Additionally, the spatial attributes are also scored higher, resulting in the overall CSA score of 2.68, which is 6.59% higher than seagrass. | |
| Littoral mud (A2.3) | 1.89 | 1.96 | 2.72 | 64.15 | Littoral muddy sediments consequence attributes are scored the same as littoral coarse sediments, mixed sediments, and littoral sand. The spatial overlap is like other inshore sedimentary habitats, but it is much more intensely fished, raising the SAR score. The overall CSA risk score is 0.94% higher than sublittoral mud. | |
| Sublittoral coarse sediment (A5.1) | 2.11 | 2.08 | 2.96 | 69.90 | Sublittoral coarse sediments consequence score is scored like sublittoral mud. The spatial overlap is similar to other littoral and sublittoral sediments but the SAR attribute is scored as one of the highest habitats. The overall CSA risk score is 1.10% higher than saltmarsh. |  |
| Coastal saltmarshes and saline reedbeds (A2.5) | 2.22 | 2.24 | 3.16 | 74.43 | Saltmarsh consequence attributes are scored like other inshore habitats, but they have a much higher regeneration time, and unlike unconsolidated sediments, lightly consolidated sediments get a slightly lower substratum hardness attribute score. The overall consequence score is the highest across all habitat types. The spatial overlap is scored quite highly, indicating 31% of the saltmarsh within the MMO region falls within the southern North Sea fished area. In addition, the SAR attribute is high, although this is likely a result of the spatial resolution of the fishing pressure data and not that fishing is taking place in these habitats but close to (the fishing pressure data has a c-square resolution of 0.05). The CSA risk score is 4.65% higher than littoral mud. | |
| Biogenic reefs (A2.7) | 2.00 | 2.47 | 3.18 | 74.89 | Biogenic reefs are scored like other sublittoral habitats, including of regeneration biota using MSC FCP v2.2. PF12 (using surrogate when data are not available). The lower score on consequence is due to a difference in scoring on the Substratum hardness attribute where biogenic reefs are soft (lightly consolidated, weathered or biogenic) with a score of 2 compared to sediments which are unconsolidated and awarded a score of 3. Partnered with one of the highest spatial scores, the CSA risk score is 3^rd^ highest of all habitats assessed in the study area, 4.99% higher than sublittoral coarse sediments. | |
| Sublittoral mixed sediments (A5.4) | 2.11 | 2.47 | 3.25 | 76.57 | Sublittoral coarse sediments consequence score is scored like sublittoral mud and coarse sediments. Spatial overlap is lower compared to biogenic reefs, but the SAR attribute is higher, indicating a higher fishing intensity. Coupled together the overall CSA risk score is 1.68% higher than biogenic reefs. | |
| Sublittoral sand (A5.2) | 2.11 | 2.82 | 3.53 | 83.14 | Sublittoral coarse sediments consequence score is scored like all other sublittoral sediments. Sublittoral sand also has the highest spatial overlap (67.8%) and SAR (>1). The overall CSA risk score is 6.57% higher than sublittoral mixed sediments. |  |

Table 11: Final CSA carbon extension scores for each habitat type (scoring element) in the southern North Sea case study. Habitats are presented in order of CSA risk score, lowest to highest.

| **Scoring element** | **Consequence score** | **Spatial score** | **CSA risk score** | **% of maximum CSA risk score (4.24)** | **Explanation of CSA risk score** |
| --- | --- | --- | --- | --- | --- |
| Littoral sand, muddy sand (A2.2) | 1.73 | 1.31 | 2.17 | 51.14 | All attributes are score the same under the CSA Carbon extension (CCSA) as the MSC CSA, the only difference in scoring therefore is under the consequence score due to the addition of Carbon attributes, The overall CSA score has decreased by 3.62% under the CCSA compared to MSC CSA. |
| Littoral rock and other hard substrata (A1.1) | 1.60 | 1.44 | 2.16 | 50.75 | The overall CSA score has increased by 2.61% under the CCSA compared to MSC CSA and is 0.15% higher than littoral sand. |
| Sublittoral mud (A5.3) | 1.87 | 1.65 | 2.49 | 58.82 | The overall CSA score has decreased by 4.39% under the CCSA compared to MSC CSA and is 7.56% higher than littoral sand. |
| Littoral sediments dominated by aquatic angiosperms (A2.6) | 2.40 | 1.14 | 2.66 | 62.63 | The overall CSA score has increased by 3.78% under the CCSA compared to MSC CSA and is 2.10% higher than sublittoral mud. |
| Littoral mud (A2.3) | 1.91 | 1.96 | 2.69 | 64.55 | The overall CSA score has increased by 0.02% under the CCSA compared to MSC CSA and is 3.77% higher than seagrass. |
| Coastal saltmarshes and saline reedbeds (A2.5) | 2.38 | 2.24 | 3.27 | 77.07 | The overall CSA score has increased by 2.71% under the CCSA compared to MSC CSA and is 7.34% higher than littoral mud. |
| Sublittoral sand (A5.2) | 1.81 | 2.82 | 3.36 | 79.15 | The overall CSA score has decreased by 4.39% under the CCSA compared to MSC CSA and is 7.24% higher than saltmarsh. |

**References**

67. Eigaard OR, Bastardie F, Breen M, Dinesen GE, Hintzen NT, Laffargue P, et al. Estimating seabed pressure from demersal trawls, seines, and dredges based on gear design and dimensions. ICES Journal of Marine Science. 2016 Jan 1;73:i27–43.

68. Lewis P. Quantifying intertidal canopy-forming macroalgal production, extent, degradation, and blue carbon potential DECLARATION OF WORK. 2020.

69. Smale DA, Pessarrodona A, King N, Burrows MT, Yunnie A, Vance T, et al. environmental factors influencing primary productivity of the forest-forming kelp Laminaria hyperborea in the northeast Atlantic. Nature [Internet]. 2020 [cited 2022 Apr 13];10(12161). Available from: https://doi.org/10.1038/s41598-020-69238-x

70. King NG, Moore PJ, Pessarrodona · Albert, Michael ·, Burrows T, Porter J, et al. Ecological performance differs between range centre and trailing edge populations of a cold-water kelp: implications for estimating net primary productivity. Mar Biol [Internet]. 2020 [cited 2022 Apr 13];167:137. Available from: https://doi.org/10.1007/s00227-020-03743-5

71. Duarte CM, Middelburg JJ, Caraco N. Major role of marine vegetation on the oceanic carbon cycle. Biogeosciences [Internet]. 2005 [cited 2022 May 11];2:1–8. Available from: www.biogeosciences.net/bg/2/1/

72. Adams CA, Andrews JE, Jickells T. Nitrous oxide and methane fluxes vs. carbon, nitrogen and phosphorous burial in new intertidal and saltmarsh sediments. Science of the Total Environment. 2012 Sep 15;434:240–51.

73. Potouroglou M. Assessing the Role of Intertidal Seagrasses as Coastal Carbon Sinks in Scotland. 2017;

74. Thornton DCO, Dong LF, Underwood GJC, Nedwell DB. Factors affecting microphytobenthic biomass, species composition and production in the Colne Estuary (UK). Aquatic Microbial Ecology. 2002 Apr 16;27(3):285–300.

75. Trimmer M, Nedwell DB, Sivyer DB, Malcolm SJ. Nitrogen fluxes through the lower estuary of the river Great Ouse, England: the role of the bottom sediments. Mar Ecol Prog Ser. 1998;163:109–24.

76. Callaway JC, DRD, and PWH: Chernobyl 137Cs used to determine sediment accretion rates at selected northern European coastal wetlands, Limnol. Oceanography. 1996;41:444–50.

77. Beaumont NJ, Jones L, Garbutt A, Hansom JD, Toberman M. The value of carbon sequestration and storage in coastal habitats. Estuar Coast Shelf Sci [Internet]. 2014 [cited 2022 May 11];137(1):32–40. Available from: http://dx.doi.org/10.1016/j.ecss.2013.11.022

78. Ford H, Garbutt A, Jones L, Jones DL. Methane, carbon dioxide and nitrous oxide fluxes from a temperate salt marsh: Grazing management does not alter Global Warming Potential. Estuar Coast Shelf Sci [Internet]. 2012 Nov 10 [cited 2022 Apr 14];113:182–91. Available from: http://dx.doi.org/10.1016/j.ecss.2012.08.002

79. Burden A, Garbutt RA, Evans CD, Jones DL, Cooper DM. Carbon sequestration and biogeochemical cycling in a saltmarsh subject to coastal managed realignment. Estuar Coast Shelf Sci [Internet]. 2013 Mar 20 [cited 2022 Apr 14];120:12–20. Available from: http://dx.doi.org/10.1016/j.ecss.2013.01.014

80. Gregg R, Elias J, Alonso I, Crosher I, Muto P, Morecroft M. Carbon storage and sequestration by habitat: a review of the evidence (second edition). 2021;

81. Burden A, Garbutt A, Evans CD. Effect of restoration on saltmarsh carbon accumulation in Eastern England. Biol Lett [Internet]. 2019 Jan 1 [cited 2022 Apr 14];15(1). Available from: https://royalsocietypublishing.org/doi/full/10.1098/rsbl.2018.0773

82. Ford H, Garbutt A, Duggan-Edwards M, Pagès JF, Harvey R, Ladd C, et al. Large-scale predictions of salt-marsh carbon stock based on simple observations of plant community and soil type. Biogeosciences. 2019 Jan 25;16(2):425–36.

83. Chmura GL ASCDLJ. Global carbon sequestration in tidal, saline wetland soils. Global Biogeochem Cycles. 2003;17.

84. Cannell M MRHK al. National inventories of terrestrial carbon sources and sinks: the UK experience. Clim Change. 1999;42:505–30.

85. Lima M do AC, Ward RD, Joyce CB. Environmental drivers of sediment carbon storage in temperate seagrass meadows. Hydrobiologia 2019 847:7 [Internet]. 2019 Dec 21 [cited 2022 Apr 14];847(7):1773–92. Available from: https://link.springer.com/article/10.1007/s10750-019-04153-5

86. Röhr ME HMBJk al. Blue carbon storage capacity of temperate eelgrass (Zostera marina) meadows. Global Biogeochem Cycles. 2018;32:1457–75.

87. Green A CMJP. Variability of UK seagrass sediment carbon: Implications for blue carbon estimates and marine conservation management. PLoS One. 2018;13.

88. Kirwan MLTS. Coastal marsh response to historical and future sea-level acceleration. Quat Sci Rev. 2009;28(17):1801–8.

89. A. B. Novak, M. C. Pelletier, P. Colarusso, J. Simpson, M. N. Gutierrez, A. Arias-Ortiz, et al. Factors Influencing Carbon Stocks and Accumulation Rates in Eelgrass Meadows Across New England, USA. Estuaries and Coasts. 2020;43:2076–91.

90. Sand-Jensen K. Biomass, net production and growth dynamics in an eelgrass (Zostera marina L.) population in Vellerup Vig, Denmark. Ophelia. 1975;14(1–2):185–201.

91. Godshalk GLWR. Decomposition of aquatic angiosperms. III. Zostera marina L. and a conceptual model of decomposition. Aquat Bot. 1978;5:329–54.

92. de Haas H BWVWT. Recent sedimentation and organic carbon burial in a shelf sea: The North Sea. Mar Geol. 1997;144:131–46.

93. Queirós AM SNWS al. Connected macroalgal-sediment systems: blue carbon and food webs in the deep coastal ocean. Ecol Monogr. 2019;89:1–21.
