# Supplementary material for: Adapting the marine stewardship council’s risk-based framework to assess the impact of towed bottom fishing gear on blue carbon habitats
Source: PLoS One. 2023 Nov 16;18(11):e0288484. doi: 10.1371/journal.pone.0288484 (PMC10653409; doi:10.1371/journal.pone.0288484)
Supplement: Attachment — Submitted filename: Response to Reviewers.docx [file pone.0288484.s003.docx]

Herriot-Watt University

The Lyell Centre

Research Avenue South

Edinburgh

EH14 4AP

Dear Editor,

We are pleased to submit our revised manuscript entitled “**Adapting the Marine Stewardship Council’s Risk-Based Framework to assess the impact of towed bottom fishing gear on blue carbon habitats**” for consideration as a publication in PLOS One.

This research manuscript offers novel insights into the management of blue carbon and will be of interest to your academic readership, civil society, and government policymakers and regulators regarding reforms to seabed management. The drive to net-zero carbon to meet International Panel for Climate Change (IPCC) emission targets, and increased efforts to mitigate the impacts of climate change have recently begun focusing attention on aquatic carbon storage. Yet, guidance on how to incorporate carbon considerations into existing fisheries management measures is absent from the available carbon literature. The key highlights from our study are: -

- The Consequence Spatial Analysis (CSA) carbon extension developed herein is the first attempt made to incorporate carbon indicators into the existing risk-based framework used by the Marine Stewardship Council (MSC) to evaluate fisheries sustainability.
- We demonstrate how the CSA carbon extension can be used to inform the sustainable, climate-conscious development of fisheries.
- To develop the CSA carbon extension, the paper comprehensively summarises published Organic Carbon stock and accumulation estimates calculated for habitats within the UK Economic Exclusion Zone.
- The CSA carbon extension is tested using a southern North Sea case study and the results presented highlight which marine habitats are most at risk to towed bottom fishing gear in the region.
- The results demonstrate that the inclusion of carbon in the MSC risk-based framework would produce different risk scores that are used to define sustainable practices through fisheries certification.

The original manuscript has been revised in response to peer review and editorial comments made, the suggested action and actions taken are summarised on the next page. All changes have been completed in tracked changes and submitted along with a clean copy of the manuscript.

The authors confirm the paper is in accordance with the remit of the journal and provides a framework for analysing the risk fishing gears pose to blue carbon, which contributes to a better understanding of the fishing sector and management approaches.

This paper is all original research carried out by the authors from 2021-present and collaboratively developed. The manuscript has not been published, or accepted for publication, nor is it under consideration elsewhere.

We look forward to hearing from you and are happy to provide additional information as necessary.

Best regards,

Kate Morris

| **Reviewer** | **Reviewer comment** | **Authors action** | **Summary of changes made and why OR why not** |
| --- | --- | --- | --- |
| Reviewer 1 | My comments on the manuscript on the whole are relatively minor. However, I am skeptical of the CSA approach given some of the scoring, and the way it is mapped spatially. I accept that for risk assessments / models that simplifications and assumptions are inevitable, and I acknowledge that CSA has been developed by others (I’m not tasked with reviewing the CSA here). However, I think that acknowledgement of some of the (what I perceive to be key) limitations and how it affects the authors' interpretations would be useful. | Accepted – paper revisions made | Thank you for your comprehensive feedback. We agree that the limitations of the study could have been outlined better, and what these limitations mean for scoring outcomes and interpretations. We have added text to clarify both the limitations of the CSA method and the consequences for the results. This also addresses some of your other comments below, where specific line numbers are provided to indicate where changes have been made. |
| Reviewer 1 | If I’ve understood correctly, CSA scores are calculated for the habitat type across the study area. That is, you have a risk score from 1 (low risk) to 4 (high risk) for each habitat type (irrespective of the extent of spatial distribution, noting your SAR can vary spatially). Your SCORE IS SCALE DEPENDENT? That is, if your assessment was undertaken at different spatial scales (let’s say a quarter of your study area), you might get different risk scores for each habitat type, but this isn’t considered here. If my interpretation is correct, then I think you need to caveat this. | Clarification provided - paper revisions made | Yes, the CSA results produced are specific to vessels under assessment (>12m deploying Mobile Bottom Contacting Gear in the Southern North Sea), and for the defined habitat types across the assessment area (the Southern North Sea). Each habitat type (termed a scoring element) is scored as a proportion of the fished area under assessment, within the context of the whole managed area.  The CSA score produced is specific to the fishery under assessment and would therefore vary depending on the fishery being assessed.  The MSC CSA and CCSA were developed to allow individual fishers to assess their own impact on different habitat types. The southern North Sea was selected purely to test how to add carbon attributes into the MSC CSA, and if the newly developed CCSA changes the results of the CSA.  We have made this clearer in the paper on lines 553-555, 556 and 565. |
| Reviewer 1 | Secondly, the authors map risk spatially, but noting that there is no variation spatially by habitat type in the current assessment. I think this needs to be made very clear, and you could also point to other assessment/methods that do allow for spatial risk to vary by habitat. For example, the RBS method which you cite (not a risk assessment per se but has been used in risk assessments) does vary spatially within habitat types because the benthic status is calculated from the distribution of bottom fishing swept area | Accepted – paper revisions made | Yes, for individual habitat types, the risk score generated is assumed to be equal across the southern North Sea case study area.  We have updated the discussion to reflect this limitation in assessment methodology (lines 555; 622-626). |
| Reviewer 1 | Thirdly, I’m somewhat confused that Sublittoral mixed sediments and sublittoral sands have a higher consequence score than most (including rocky reefs and Biogenic reefs!). This is irrespective of the SAR. I understand that you haven’t come up with these estimates, and that these have been extensively developed by others, but I think it would be worthwhile making it clear somewhere why the scores are what they are and why they make sense, because intuitively as an ecologist they don’t to me. This doesn’t need to be long, e.g., you could include a short justification / driver for each habitat type in the appendix next to the scores? | Accepted – paper revisions made | An additional column has been added to Table 10 and Table 11 to provide an explanation of the CSA scores and why they may differ between habitat types.  Note: When reviewing table 11 in response to this comment, the authors realised that the saltmarsh scoring element was absent. This has been updated along with the correct habitat productivity scores (i.e., where the saltmarsh element is included in how the carbon attributes scores are calculated). |
| Reviewer 1 | Finally, the use of EUNIS at the broad scale is acknowledged as a limitation in the discussion. But I think more of this needs to be brought out. For example, highlighting the sheer number of possible biological communities that can be associated with each of the level 3 habitats. I.e., the risk, must be different between reefs with soft corals vs those with mixed hydroid / bryozoan turf with encrusting pomatoceros worms (a very common habitat in some parts of the UK). I think that the spatial variation in these biological communities would greatly influence the spatial representation of risk and should be explicitly acknowledged in the discussion. I think the authors can again point to methods may address these limitations in the future and how carbon attributes will vary between these communities (i.e. point to future work, no change to approach here). | Accepted – paper revisions made | Thank you for your comment. We have added text to lines 606-618 to highlight this limitation of available habitat data, and how the MSC CSA / CCSA methodology deals with the interaction between habitat and fishing pressure data. |
| Reviewer 1 | Introduction: Check heading format / numbering for publication. | Accepted – paper revisions made | Thank-you. Kate |
| Reviewer 1 | L 173: Can you provide some examples of the aforementioned examples applied to real world fisheries management? E.g., see the joint Australian / New Zealand Bottom Fisheries Impact Assessment: <https://www.sprfmo.int/science/bottom-fishing/> But it would be good to provide a couple more examples given you state risk assessments have supported “many fisheries management systems” |  | From the UK we have a recent example from the Seafish Authority, which is using an Ecological Risk Assessment (including use of a Consequence Spatial Analysis) in marine plans - [here](https://www.seafish.org/about-us/news-blogs/a-tool-to-help-manage-southwest-england-fisheries?_cldee=TKxBP8xfEjkJFqpbASFOW65lytzWGHsQMbHT9R6idoRHcTRC132a0y1q0XoCRwxi&recipientid=contact-b79427402294ed11aad1000d3a87575a-172e9755f19742a2aeb66d80e14eaab7&utm_source=ClickDimensions&utm_medium=email&utm_campaign=Fisheries%20Management%20Innovation%20Group&esid=e19f3626-4cd9-ed11-a7c7-000d3a872ec0) |
| Reviewer 1 | Figure 1. Consider (but not essential) relabelling the graticules to indicate cardinal points rather than – or + signs. I.e., 10°W (rather than -10); you can also remove the North arrow and scale bar if you have graticules on the map. Both of these points are personal preference and I’ll leave it to the authors / editor to decide. | Accepted – paper revisions made | The figure has been updated, and where relevant to other figures, they have been amended too. |
| Reviewer 1 | L 212-214: it is not clear where you are getting level 4 EUNIS classification from? Where are the maps for these? My search of reference 36 only shows EUNIS level 3. Can you include the map in the supplementary materials or a link to a GIS map? | Clarification provided – paper revisions made | Apologies, this is an oversight the habitat map was updated and the level 4 EUNIS classifications were retracted by one of the contributing organisations however this text in the paper was not updated. The only changes to the map are where habitat classifications were mixed and redefined to a single EUNIS category (all being a EUNIS level 3). This has been updated on line 215. |
| Reviewer 1 | General comment about EUNIS habitats: These are very broadscale. i.e., 0 – 25m for littoral kelp dominated habitats is not very realistic for many parts of the UK; just because there is rock at 20m does not mean there is L digitata (suggest this could be better estimated with some kind of estimate of turbidity / PAR to the seafloor estimate if available – or pointed to in the discussion as a future direction). The overestimate of kelp is acknowledged in the discussion, but see my comments about more emphasis on how scores may change with other biological communities accounted for (and ideally how their spatial distribution can also affect spatial distribution of risk). | Partially accepted – no paper revisions made | The authors do agree that more granular habitat mapping is required for all habitat types, especially kelp, macroalgae and more vulnerable habitats such as corals. But it was not within the purview of this study to provide a more accurate estimate of kelp distribution. The limitations of making such an assumption are acknowledged and we have encouraged the CCSA be replicated once better habitat and fishing pressure data become available. |
| Reviewer 1 | L242: Please provide the grid size of c-square. | Accepted – paper revisions made | ICES C-square is 0.05, this information has been added to line 241. |
| Reviewer 1 | L246: formatting of reference | Clarification provided – paper revisions made | We can’t find the reference referred to on 246 however a few others have been picked up and corrected. |
| Reviewer 1 | L294: Looking at Fig 6 in appendix, you can you get SAR > 1 (at least some areas are fished more than once per year). If so, how do you account for this in the risk assessment? I.e., should the CSA score be 1.5 or 2 for SAR > 1? Whether such a change would result in a different risk outcome is the key question. If not, then ok to leave as 1, but if this particular score has a high influence on the final risk assessment, then worth considering where SAR > 1 should have a different risk score. Either way, I think this should be considered in greater detail the discussion. | Clarification provided – No paper revisions made | This was considered at length by the co-authors, but the MSC CSA applies a discrete risk ranking method, therefore, it was concluded that scoring the SAR using a quantile rank (as seen in Figure A6) was most appropriate. Changing how SAR attribute scores are calculated would only affect a small proportion of the fished area, which is above >0.57 SAR.  Furthermore, it was decided that this way, the SAR attribute scores reflect the relationship observed between fishing pressure, depletion, and recovery of benthic communities (Figure 3 (A and C) Hiddink et al., 2017). |
| Reviewer 1 | L296: are there risks associated with cumulative impacts resulting in a shift in dominant biota. I.e., the kelp does not recover? | Clarification provided – no paper revisions made | Cumulative impacts or community shifts are not considered in the MCS CSA or CCSA, both are designed to provide a snapshot at a specific time point of the risk to current benthic communities. |
| Reviewer 1 | L384: I think the authors need to be careful here. By normalising the scores, they are effectively saying that CSA score (risk) MUST go from low – high and that scores are relative to each other. I don’t have an issue with the relative to each other part of the assumption. What I’d be most concerned about is stating that there is a low CSA score for some habitats when they are all at moderate – high risk (i.e., there are no “low” carbon capacity attributes). I think this possible limitation needs to be acknowledged and the authors need to justify why they think this normalisation (rather than a scoring based on evidence / expert knowledge) is the best approach and does not produce incorrect CSA scores. | Accepted – paper revisions made | The carbon attribute scores have been calculated based on existing values of stock and accumulation for the different habitat types in the UK EEZ. We appreciate the comment made here, so we added an additional tab in the scoring Excel file to see how a habitat type with a default 0 (dummy) value for carbon stock and accumulation would affect the lower carbon capacity attributes. For Carbon Stock, rock went from 1 to 1.04 and for Carbon Accumulation, sublittoral sand went from 1 to 1.17. Although these differences aren’t huge, if we had more data for other habitats, it would allow us to add them to the CCSA and they would still fall on a sensible scale and the results of the present study would still be accurate (line 377-381).  The CCSA results presented have been updated in the relevant tables and figures.  We have also amended the wording when referring to the carbon scores - as in use "lower" rather than "low" in lines 401 and 408-9. |
| Reviewer 1 | L421 – I don’t understand how or why CSA aims to capture how “economically advantageous the habitat is to fishing activities” and how that feeds into a risk assessment? Suggest clarifying. | Clarification provided –paper revisions made | This wording is adapted from Hobday *et al*, 1968 and MSCs FCP v2.2. Specifically, where it is considered “*Rugged substratum is less accessible to mobile gears. Steeply sloping seabed is less accessible to mobile gears*” (Hobday *et al*, 1968). So, the substratum attribute aims to capture how economically advantageous or attractive a fishing ground is based on its topology. Line 432 has been amended to “*aim to capture the* *appropriateness of the habitat to fishing activities*”. |
| Reviewer 1 | Section 2.1 (first section of results) – I suggest this is moved to the methods. | Clarification provided –no paper revisions made | As the incorporation of carbon attributes into the CSA risk assessment is the primary output from this investigation, we feel it is appropriate for this final stage to be included in the results section of the manuscript. |
| Reviewer 1 | Fig 4. It might be the low resolution of the figures in the draft manuscript, but suggest that you could make Null values clearer (either diff colour, or with hashing, or something). | Accepted – paper revisions made | Thank you, we have updated Figure 4 and clarified the Null values by marking these areas with a dark-crosshatch pattern. |
| Reviewer 1 | L 601: consider providing examples of methods which would allow SAR to be calculated inshore (e.g., using Fisher knowledge, or IFCA monitoring): Turner, R.A., Polunin, N.V.C., and Stead, S.M. (2015). Mapping inshore fisheries: Comparing observed and perceived distributions of pot fishing activity in Northumberland. Marine Policy 51, 173-181. Szostek, C.L., Murray, L.G., Bell, E., and Kaiser, M.J. (2017). Filling the gap: Using fishers’ knowledge to map the extent and intensity of fishing activity. Marine Environmental Research 129, 329-346. | Accepted – paper revisions made | Thank you for these additional references. These have been added to the paper in lines 621. |
| Reviewer 2 | The study is a useful extension of the MSCs risk assessment framework. It would be helpful for MSC assessment improvement and in giving a more fulsome picture of certified fishery sustainability if this new benthic carbon disturbance risk analysis methodology becomes a new plug-in to MSC assessments in future.  Further to that I have minor editorial comments that you’ll no doubt pick up in your final edit. | Accepted – no revisions made | Thank you for your review of this paper and the comments provided.  Unfortunately, we’ve not been provided with your minor editorial comments, but we hope in this 2^nd^ review most if not all, have been corrected. |
| Editorial review | Please ensure that your manuscript meets PLOS ONE's style requirements, including those for file naming. The PLOS ONE style templates can be found at <https://journals.plos.org/plosone/s/file?id=wjVg/PLOSOne_formatting_sample_main_body.pdf> and  <https://journals.plos.org/plosone/s/file?id=ba62/PLOSOne_formatting_sample_title_authors_affiliations.pdf> | Accepted – No revisions made | Thank-you, the requirements have been double checked. |
| Editorial review | We note that you have stated that you will provide repository information for your data at acceptance. Should your manuscript be accepted for publication, we will hold it until you provide the relevant accession numbers or DOIs necessary to access your data. If you wish to make changes to your Data Availability statement, please describe these changes in your cover letter and we will update your Data Availability statement to reflect the information you provide | Accepted – No changes made | No changes to the data availability statement have been made. Both the fishing pressure data, habitat data and scoring excel file will be made available on publication. |
| Editorial review | We note that Figure (1, 4 and 5) in your submission contain copyrighted images. All PLOS content is published under the Creative Commons Attribution License (CC BY 4.0), which means that the manuscript, images, and Supporting Information files will be freely available online, and any third party is permitted to access, download, copy, distribute, and use these materials in any way, even commercially, with proper attribution. For more information, see our copyright guidelines: <http://journals.plos.org/plosone/s/licenses-and-copyright>. | Clarification provided – no paper revisions made | All figures have been produced in ArcGIS and are original works. Content has not been taken from any third party unless layers from published literature have been used. |
|  | Please review your reference list to ensure that it is complete and correct. If you have cited papers that have been retracted, please include the rationale for doing so in the manuscript text or remove these references and replace them with relevant current references. Any changes to the reference list should be mentioned in the rebuttal letter that accompanies your revised manuscript. If you need to cite a retracted article, indicate the article’s retracted status in the References list and also include a citation and full reference for the retraction notice. | Not accepted – no paper revisions made | The article does not refer to any retracted journal papers. |

References:

Hiddink, J. G., Jennings, S., Sciberras, M., Szostek, C. L., Hughes, K. M., Ellis, N., Rijnsdorp, A. D., McConnaughey, R. A., Mazor, T., Hilborn, R., Collie, J. S., Pitcher, C. R., Amoroso, R. O., Parma, A. M., Suuronen, P., & Kaiser, M. J. (2017). Global analysis of depletion and recovery of seabed biota after bottom trawling disturbance. *Proceedings of the National Academy of Sciences of the United States of America*, *114*(31), 8301–8306. https://doi.org/10.1073/PNAS.1618858114
